# Applications of Biosurfactants in the Petroleum Industry and the Remediation of Oil Spills

**DOI:** 10.3390/ijms150712523

**Published:** 2014-07-15

**Authors:** Rita de Cássia F. S. Silva, Darne G. Almeida, Raquel D. Rufino, Juliana M. Luna, Valdemir A. Santos, Leonie Asfora Sarubbo

**Affiliations:** 1 Post-Graduate Program in Biotechnology, Federal Rural University of Pernambuco, CEP 52.171-900 Recife, PE, Brazil; E-Mails: ritaf78@gmail.com (R.C.F.S.S.); darne@buenomak.com.br (D.G.A.); 2 Center for Management of Technology and Innovation—CGTI, Rua da Praia, n.11, São José, CEP 50.020-550 Recife, PE, Brazil; E-Mails: raqueldrufino@yahoo.com.br (R.D.R.); julianamouraluna@gmail.com (J.M.L.); valdemir.alexandre@hotmail.com (V.A.S.); 3 Center for Sciences and Technology, Catholic University of Pernambuco, Rua do Príncipe, n. 526, Boa Vista, CEP 50.050-900 Recife, PE, Brazil

**Keywords:** surface tension, industrial wastes, patents, sustainable technologies

## Abstract

Petroleum hydrocarbons are important energy resources. However, petroleum is also a major pollutant of the environment. Contamination by oil and oil products has caused serious harm, and increasing attention has been paid to the development and implementation of innovative technologies for the removal of these contaminants. Biosurfactants have been extensively used in the remediation of water and soil, as well as in the main stages of the oil production chain, such as extraction, transportation, and storage. This diversity of applications is mainly due to advantages such as biodegradability, low toxicity and better functionality under extreme conditions in comparison to synthetic counterparts. Moreover, biosurfactants can be obtained with the use of agro-industrial waste as substrate, which helps reduce overall production costs. The present review describes the potential applications of biosurfactants in the oil industry and the remediation of environmental pollution caused by oil spills.

## 1. Introduction

Petroleum is one of the most important energy resources and a raw material of the chemical industry. The world depends on oil and the use of oil as fuel has contributed to intensive economic development. Although petrochemical plants and oil refineries are beneficial to society, they produce a large amount of hazardous waste. Moreover, oil spills during exploration, transportation, and refining, have caused serious environmental problems [1,2,3,4,5,6]. 

The remediation of contaminated sites can be achieved by physicochemical or biological methods. Conventional physicochemical methods can rapidly remove the majority of spilled oil, but, in most cases, removal simply transfers contaminants from one environmental medium to another and can even produce toxic byproducts. Moreover, crude oil cannot be completely cleaned up with physicochemical methods. Thus, more attention is being given to biological alternatives [7,8]. Biosurfactants play an important role in remediation processes due to their efficacy as dispersion and remediation agents as well as their environmentally friendly characteristics, such as low toxicity and high biodegradability [9]. Indeed, biosurfactants have applications in different industrial processes as well as possible novel uses in the future and are expected to become known as multifunctional materials of the 21st century [5]. Currently, the major market for biosurfactants is the petroleum industry, in which these compounds can be used in the cleanup of oils spills, the removal of oil residue from storage tanks, microbial-enhanced oil recovery, and the bioremediation of soil and water [3]. 

The diverse structures of biosurfactants have useful properties with industrial potential. The production of surfactants by microorganisms is performed to enhance both the bioavailability of nearly inaccessible substrates and survival under conditions of low moisture. Biosurfactant production generally requires a hydrophobic and hydrophilic carbon source in the culture medium. The process is economically and environmentally attractive when waste products are used [10,11]. 

This review addresses the potential roles and applications of biosurfactants, focusing on the petroleum industry and bioremediation processes. The key features of the microbial biosynthesis of biosurfactants, their physicochemical and bioactive properties and the use of industrial wastes as cost-effective alternatives for biosurfactant production are also summarized.

## 2. Biosurfactants

The advance of sustainable technologies has driven the search for natural, biodegradable compounds to remediate sites contaminated by hydrocarbons. This has led to the discovery of surfactants of a natural origin. Most of these surfactants are synthesized by living organisms, such as, saponins produced by plants, glycolipids produced by microorganisms, and bile salts produced by animals. Compounds with surfactant properties produced by microorganisms are denominated biosurfactants [2,12].

Biosurfactants are mainly produced by aerobic microorganisms in aqueous media with a carbon source feedstock, such as carbohydrates, hydrocarbons, fats, and oils. It is believed that biosurfactants are secreted into the culture medium to assist in the growth of the microorganism by facilitating the translocation of insoluble substrates across cell membranes [13]. These compounds have amphipathic molecules with hydrophobic and hydrophilic portions that act between fluids of different polarities (oil/water and water/oil), allowing access to hydrophobic substrates and causing a reduction in surface tension, an increase in the area of contact of insoluble compounds (such as hydrocarbons) and the enhancement of the mobility, bioavailability, and biodegradation of such compounds [12]. The lipophilic moiety can be a protein or peptide with a high proportion of hydrophobic side chains or a hydrocarbon chain of a fatty acid with 10 to 18 carbon atoms, although fatty acids with a higher molecular weight have been reported. The hydrophilic moiety can be an ester, hydroxy, phosphate, carboxylate group, or sugar [13]. Biosurfactants are generally classified into low molecular-mass molecules, which efficiently lower surface and interfacial tensions, and high molecular-mass polymers, which are more effective as emulsion-stabilizing agents. The major classes of low-mass surfactants are glycolipids, lipopeptides, and phospholipids, whereas high-mass surfactants include polymeric and particulate surfactants [14].

Biosurfactants offer a number of advantages over chemical surfactants, such as biodegradability due to their simple chemical structure, environmental compatibility, low toxicity, which allows use in the cosmetic, pharmaceutical and food industry, high selectivity due to presence of specific functional groups, allowing specificity in the detoxification of specific pollutants, and activity under conditions of extreme temperatures, pH and salinity [14,15]. These traits contribute to the applicability of biosurfactants in different industries [16,17].

The fact that biosurfactants are characterized by a vast structural diversity and display a broad range of properties may explain why this group of molecules continues to pique scientific interest [5,16,18]. Due to their diverse industrial applications, many authors have filed for patents on biosurfactants. Indeed, patents have been issued for biosurfactant producing microbes, especially *Pseudomonas* spp., *Acinetobacter* spp., *Bacillus* spp., and *Candida* spp., types of biosurfactant, the production process and industrial applications [19]. Table 1, adapted from Sachdev and Cameotra [19], lists some of the important patents issued in recent years.

The economics of biosurfactant production merit particular attention. The total production of surfactants in 2012 was ~12 million tons, only 3.5 million tons of which were biosurfactants. Moreover, revenues from the bio-based portion of the market were US$ 6.588 million [13]. However, the focus on sustainability and new environmental legislation has led to the search for natural surfactants as alternatives to existing products [13]. Industries are currently seeking to replace some or all chemical surfactants with sustainable biosurfactants [5], but the high production cost is a major drawback. A key factor governing the success of biosurfactant production is the development of an economical process that uses low-cost materials and offers high yield. Indeed, the choice of a low-cost substrate is important to the overall economics, as the substrate accounts for up to 50% of the final production cost. Fortunately, biosurfactants can be produced with economical, renewable resources, such as vegetable oils, distillery waste, and dairy waste [20]. 

Essentially agricultural countries, such as Brazil, have easy access to agro-industrial byproducts. Table 2 lists wastes and byproducts studied by Brazilian researchers to help reduce the cost of biosurfactant production.

**Table 1:** Patents on biosurfactants produced by microorganisms.

Microorganism/Type of Biosurfactant	Patent Holder	Title of Patent	Publication No.	Publication Date
Sophorolipid producer	Borzeix F	Sophorolipids as stimulating agent of dermal fibroblast metabolism	US 6057302 A	2 May 2000
Sophorolipid producer	Borzeix F, Concaix	Use of sophorolipids comprising diacetyl lactones as agent for stimulating skin fibroblast metabolism	US 6596265 B1	22 July 2003
New strains of hydrocarbon-degrading bacteria capable of producing biosurfactants	Robin L. Brigmon, Sandra Story, Denis Altman, Christopher J. Berry	Surfactant biocatalyst for remediation of recalcitrant organics and heavy metals	PI 0519962-0 A2	28 June 2005
Sophorolipid producer	Gross RA, Shah V, Doncel GF	Spermicidal and virucidal properties of various forms of sophorolipids	WO 2005089522 A2	29 September 2005
*C. albicans,* *C. rugosa,* *C. tropicalis,* *C. lipolytica,* *C. torulopsis*	Awada S, Spendlove R, Awada M	Microbial biosurfactants as agents for controlling pests	US 20050266036 A1	1 December 2005
*Pseudomonas aeruginosa*	Silvanito Alves Barbosa, Roberto Rodrigues De Souza	Biosurfactant production for development of biodegradable detergent	PI 1102592-1 A2	16 May 2011
Sophorolipid producer	Cox TF, Crawford RJ, Gregory LG, Hosking SL, Kotsakis	Mild to skin, foaming detergent composition	WO2011120776 A1	6 October 2011
*Streptomyces sp.*	Ana LF Porto, Eduardo F Santos, Leonie A Sarubbo	Biosurfactant and production process	PI 1105951-6 A2	28 November 2011
*Candida guilliermondii*	Leonie A Sarubbo, Valdemir A Santos, Raquel D Rufino, Juliana M Luna	Production process of biosurfactant produced by *Candida guilliermondii* using agro-industrial waste	BR102012023115	13 September 2012
*Candida bombicola* ATCC 2214	Soetaert W, De MS, Saerens K, Roelants S, Van BI	Modified sophorolipid production by yeast strains and uses	EP 2580321 A1	17 April 2013
Lipopeptide producer	X. Vecino, R. Dvesa-Rey, J.M. Cruz, A.B. Moldes	Method for separating the surfactants present in the washing liquors of corn and uses	WO2014044876 A1	27 March 2014

**Table 2:** Waste and byproducts used for biosurfactant production and respective producing microorganisms.

Waste/by-Product	Biosurfactant-Producing Microorganism	Reference
Canola waste frying oil and corn steep liquor	*Pseudomonas cepacia* CCT6659	[21]
Glycerol	*Pseudomonas aeruginosa* UCP0992	[22]
Clarified cashew apple juice	*Bacillus subtilis* LAMI005	[23]
Vinasse and waste frying oil	*Bacillus pumilus*	[24]
Cassava wastewater	*Bacillus subtilis* LB5a	[25]
Soybean oil refinery residue and corn steep liquor	*Candida sphaerica* UCP0995	[26]
Ground-nut oil refinery residue and corn steep liquor	*Candida sphaerica* UCP0995	[27]
Animal fat and corn steep liquor	*Candida lipolytica* UCP0988	[15]
Vegetable fat	*Candida glabrata* UCP1002	[28]
Waste frying oil	*Candida tropicalis* UCP0996	[29]
Molasses	*Pseudomonas aeruginosa* (P.A.)	[30]

## 3. Biosurfactant-Producing Microorganisms

A number of microorganisms, such as fungi, yeasts, and bacteria, feed on substances that are immiscible in water, producing and using a surface-active substance (biosurfactant) [3,17]. Among bacteria, the genus *Pseudomonas* is known for its capacity to produce extensive quantities of glycolipids. These biosurfactants are classified as rhamnolipids. *Bacillus subtilis* is another microorganism widely studied for biosurfactant production and is known for its efficiency in producing a lipopeptide with surface activity denominated surfactin or subtilisin [2,12,31,32,33]. *Candida* *bombicola* and *Candida* *lipolytica* are among the most commonly studied yeasts for the production of biosurfactants [13]. Table 3 offers a list microorganisms that produce biosurfactants [3,34].

**Table 3:** Main classes of biosurfactants and respective producer microorganisms.

Class/Type of Biosurfactant	Microorganisms	
**Glycolipids**		
Rhamnolipids		*Pseudomonas aeruginosa*
Sophorolipids		*Torulopsis bombicola*, *T. apícola*
Trehalolipids		*Rhodococcus erythropolis*, *Mycobacterium* sp.
**Lipopeptides and lipoproteins**		
Peptide-lipid		*Bacillus licheniformis*
Viscosin		*Pseudomonas fluorescens*
Serrawettin		*Serratia marcenscens*
Surfactin		*Bacillus subtilis*
Subtilisin		*Bacillus subtilis*
Gramicidin		*Bacillus brevis*
Polymyxin		*Bacillus polymyxia*
**Fatty acids, neutral lipids and phospholipids**		
Fatty acid		*Corynebacterium lepus*
Neutral lipids		*Nocardia erythropolis*
Phospholipids		*Thiobacillus thiooxidans*
**Polymeric surfactants**		
Emulsan		*Acinetobacter calcoaceticus*
Biodispersan		*Acinetobacter calcoaceticus*
Liposan		*Candida lipolytica*
Carbohydrate-lipid-protein		*Pseudomonas fluorescens*
Mannan-lipid-protein		*Candida tropicalis*
**Particulate surfactant**		
Vesicles		*Acinetobacter calcoaceticus*

## 4. Environmental Contamination by Oil Spills and Biosurfactant-Enhanced Remediation

The release of contaminants, such as petroleum and petroleum byproducts, into the environment is one of the main causes of global pollution and has become a focus of great concern both in industrialized and developing countries due to the broad environmental distribution in soil, groundwater, and air [18,35]. The contamination sources are diverse: accidents during fuel transportation by ships and trucks; leakage from underground storage tanks that are subject to corrosion, such as gas stations; oil extraction and processing operations; and inadequate release of waste generated by industries that use oil byproducts in the production of plastics, solvents, pharmaceuticals, and cosmetics [16,36,37,38]. Half the world’s oil production (around three billion tons/year) is transported by ship and hydrocarbon contamination levels in different marine ecosystems have increased due to accidents. The major hydrocarbon source in oceans comes from routine operations of ship washing, natural oil leakage on the sea bed, and accidents during oil exploration and transportation [2].

The media has consistently reported the leakage of thousands of tons of oil that contaminate seawater [39]. One of the most impacting spills occurred in November of 2011 on the Sedco 706 oil rig, operated by Chevron Brazil in Campos Bay (Rio de Janeiro, Brazil). A total of 5943 L leaked, covering 163 km^2^ [2]. Another of the largest oil spills in the world occurred in the Gulf of Mexico in 2010, following the explosion of an oil rig off the coast of the states of Louisiana and Mississippi (USA). After the sinking of the rig, the open ducts in the drilling area (depth of 1.5 kms) continued spewing oil into the sea for a three-month period before finally being capped. Official reports indicate the release of a thousand barrels of oil per day, with an estimated total of three to four million barrels of oil spilled, making this the largest environmental disaster in the history of the United States [6]. In July 2010, an oil spill of 1500 tons of crude oil caused serious environmental problems to the ocean and coast in Dalian, China [1]. In January 2000, more than 1.3 million L of heavy oil leaked from a refinery pipeline into Guanabara Bay in Rio de Janeiro, Brazil, causing extensive damage to preserved mangrove areas [40].

Over ten events of oil tankers with important wastes have occurred in Europe since 1967. The *Prestige* oil spill may be considered as one of the worst. The oil tanker *Prestige*, loaded with a cargo of 77,000 tons of heavy bunker oil ran into problems off the Galician coast (NW Spain) on 13 November 2002. After several days following an erratic path and spilling 19,000 tons, the tanker finally sank 130 miles west off the southern coast [41].

Large amounts of crude oil entering the marine environment, groundwater and soil can cause significant harm to resident organisms [8]. Petroleum is a hydrophobic hydrocarbon with negative effects on the structural and functional properties of cell membranes in living organisms, offering considerable risk of contamination in both marine and terrestrial ecosystems [3]. When in contact with water, oil and its byproducts spread and form a thin layer on the surface that prevents gas exchange between the air and water, blocking the passage of sunlight and impeding the respiration and photosynthesis process. Thus, hydrocarbon waste impacts phytoplankton communities, causing a fundamental breakdown of the food chain [42,43]. The potential threat to human health posed by hydrocarbons is linked to the physical and chemical properties of these compounds, which are absorbed by the skin and quickly spread through the organism if ingested or inhaled [44,45].

The most common role of biosurfactants is to enhance the dispersal of contaminants in the aqueous phase and increase the bioavailability of the hydrophobic substrate to microorganisms, with subsequent removal of such pollutants through biodegradation [12,46]. Numerous examples demonstrate the potential application of biosurfactants in environmental decontamination. Sobrinho *et al*. [27] tested a biosurfactant produced by *Candida sphaerica* for the removal of motor oil from soil and seawater and found removal rates of 75% and 92% from clay and silty soil, respectively; in tests carried out with seawater, the biosurfactant exhibited an oil spreading efficiency of 75%, demonstrating its potential for application as an adjuvant in biotechnological processes of environmental decontamination. Batista *et al*. [29] investigated the application of a biosurfactant produced by *Candida tropicalis* for removing motor oil from sand and found removal rates of 78% to 97%, demonstrating considerable potential with regard to soil bioremediation. Gusmão *et al*. [28] investigated the application of a crude biosurfactant produced by Candida glabrata UCP1002 in a soil-water-hydrophobic contaminant system and found a removal rate as high as 92.6%. Luna *et al*. [26] evaluated a new biosurfactant, denominated Lunasan, produced by *Candida sphaerica* UCP 0995. This biosurfactant removed 95% of motor oil adsorbed to sand, demonstrating considerable potential for use in bioremediation processes. The remediation of hydrocarbons contaminated soils using a cell-bound biosurfactant from *Lactobacillus pentosus* has been also described [47,48]. Table 4 offers a list of different types of biosurfactants and their producing microorganisms with potential applications in the bioremediation of oil-polluted environments [10,49].

**Table 4:** Biosurfactants, producing microorganisms and uses in the bioremediation of oil-contaminated environments.

Microorganisms	Type of Biosurfactant	Applications
*Rhodococcus erythropolis* 3C-9	Glucolipid and trehalose lipid	Oil spill cleanup operations
*Pseudomonas aeruginosa* S2	Rhamnolipid	Bioremediation of oil-contaminated sites
*Rhodococcus* sp. TW53	Lipopeptide	Bioremediation of marine oil pollution.
*R. wratislaviensis* BN38	Glycolipid	Bioremediation applications
*Bacillus subtilis* BS5	Lipopeptide	Bioremediation of hydrocarbon-contaminated sites
*Azotobacter chroococcum*	Lipopeptide	Environmental applications.
*Pseudomonas aeruginosa* BS20	Rhamnolipid	Bioremediation of hydrocarbon-contaminated sites
*Micrococcus luteus* BN56	Trehalose tetraester	Bioremediation of oil-contaminated environments
*Nocardiopsis alba* MSA10	Lipopeptide	Bioremediation
*Pseudoxanthomonas* sp. PNK-04	Rhamnolipid	Environmental applications
*Pseudomonas alcaligenes*	Rhamnolipid	Environmental applications
*Nocardiopsis lucentensis* MSA04	Glycolipid	Bioremediation of marine environment
*Calyptogena soyoae*	Mannosylerythritol lipid	Bioremediation of marine environment
*Pseudozyma hubeiensis*	Glycolipid	Bioremediation of marine oil pollution
*Pseudomonas cepacia* CCT6659	Rhamnolipid	Bioremediation of marine and soil environments
*Candida bombicola*	Sophorolipids	Environmental applications
*C. glabrata* UCP1002	Protein-carboydrate-lipid complex	Oil recovery from sand
*C. lipolytica* UCP0988	Sophorolipids	Oil recovery
*C. lipolytica* UCP0988	Sophorolipids	Oil removal
*C. sphaerica* UCP0995	Protein-carboydrate-lipid complex	Removal of oil from sand
*C. lipolytica* UCP0988	Sophorolipids	Control of environmental oil pollution
*C. sphaerica* UCP0995	Protein-carboydrate-lipid complex	Bioremediation processes
*C. glabrata* UCP1002	Protein-carboydrate-lipid complex	Oil removal
*C. guilliermondii* UCP0992	Glycolipid complex	Removal of petroleum derivate motor oil from sand
*C. tropicalis* UCP0996	Protein-carboydrate-lipid complex	Removal of petroleum and motor oil adsorbed to sand
*C. lipolytica* UCP0988	Sophorolipids	Removal of petroleum and motor oil adsorbed to sand
*C. sphaerica* UCP0995	Protein-carboydrate-lipid complex	Oil removal

## 5. Application of Biosurfactants in the Petroleum Industry

Petroleum is one of the major energy sources. The energy demand in the world indicates a 1.7% increase in the number of barrels of oil produced per year, between 2000 and 2030, while consumption is expected to reach 15.3 billion tons of oil per year. Oil reserves allow meeting the world’s demand for approximately 40 years if current levels of consumption are maintained [45,50,51]. It is therefore important to develop technologies that allow the efficient use of this resource. According to the International Energy Agency, petroleum production is steadily moving toward unconventional crude oils, such as heavy and extra-heavy oils rather than medium and light oils. Heavy and extra-heavy crude oils represent at least one half of recoverable oil resources in countries such as Canada, China, Mexico, Venezuela, and the USA [52].

In the petroleum industry, biosurfactants have been applied effectively for the exploration of heavy oil, offering advantages over their synthetic counterparts throughout the entire petroleum processing chain (extraction, transportation and storage). Biosurfactants are used in microbial-enhanced oil recovery, the cleaning of contaminated vessels and to facilitate the transportation of heavy crude oil by pipeline [53,54]. Table 5 offers a list of biosurfactant applications in the oil industry [53].

**Table 5:** Common applications of biosurfactants in the petroleum industry.

Step in Petroleum Production Chain	Applications
*Extraction*	Reservoir wettability modification
	Oil viscosity reduction
	Drilling mud
	Paraffin/asphalt deposition control
	Enhanced oil displacement
	Oil viscosity reduction
*Transportation*	Oil viscosity reduction
	Oil emulsion stabilization
	Paraffin/asphalt deposition
*Oil tank/container cleaning*	Oil viscosity reduction
	Oily sludge emulsification
	Hydrocarbon dispersion

### 5.1. Extraction of Crude Oil from Reservoirs

Several enhanced oil recovery processes are currently employed worldwide: thermal, chemical, physical, *etc*. [55]. However, these processes are very expensive as well as environmentally harmful. Thus, the search for alternative, cost-effective, eco-friendly alternatives to the chemical and thermal enhanced oil recovery methods is necessary. A number of biotechnology-based processes have been proposed to increase oil production in the current energy shortage [56]. Biosurfactants have applications in this realm, as these natural compounds improve the mobilization of hydrocarbons, thereby enhancing the recovery of crude oil from reservoirs in a process denominated microbial-enhanced oil recovery (MEOR) [57]. 

MEOR consists of the tertiary recovery of oil in which microorganisms or their metabolic products are used to recover residual oil. Microorganisms produce polymers and biosurfactants, which reduce oil-rock surface tension by diminishing the capillary forces that impede the movement of oil through the pores of rock. Biosurfactants also aid in the emulsification and breakdown of oil film in rock. MEOR involves different strategies, such as the injection of microorganisms that produce biosurfactants into the reservoir and subsequent spread in situ, the injection of nutrients into the reservoir to stimulate the growth of wild microorganisms that produce biosurfactants or the further production of biosurfactants in reactors and subsequent injection into the reservoir [31]. These processes enhance oil recovery from a depleted reservoir, thereby extending the life of the reservoir. MEOR is less-expensive in comparison to chemically-enhanced oil recovery, as microorganisms produce efficient products out of low-cost substrates or raw materials [58].

### 5.2. Transport of Crude Oil by Pipelines

Crude oil often needs to be transported over long distances from the extraction fields to refineries. The transportation of heavy and extra-heavy crude oil entails operational difficulties that limit its economic viability. The major problems are low flowability due to the high degree of viscosity and asphaltene content in heavy crude oil, which leads to inconveniences, such as the deposition of asphaltenes and/or paraffins as well as a drop in pressure that can cause plugging problems in the pipeline [52,57]. Asphaltenes precipitate in metal pipelines and in presence of ferric ions combined with acidic conditions, forming a solid denominated “asphaltene mud”, which deposits in the pipeline and obstructs the free flow of crude oil. Solvents, such as toluene and xylene, are applied to dissolve this type of mud, which increases the production cost and generates highly toxic waste residue [53]. 

A promising technology involving the production of a stable oil-in-water emulsion that facilitates oil mobility has been recently developed. Biosurfactant-based emulsifiers (bioemulsifiers) are particularly suitable for this application. Bioemulsifiers are high-molecular weight surfactants with different properties in comparison to glycolipids and lipopeptides. These products are not effective at reducing interfacial tensions, but have an excellent capacity to stabilize oil-in-water emulsions. Due to the high number of reactive groups in the molecule, bioemulsifiers bind tightly to oil droplets and form an effective barrier that prevents drop coalescence [57]. Emulsan is the most powerful bioemulsifier and has potential applications in the petroleum industry, including the formation of heavy oil-water emulsions for viscosity reduction during pipeline transport [53,57].

### 5.3. Oil Storage Tank Cleaning

Large amounts of crude oil are moved daily, distributed to refineries and placed in storage tanks. The maintenance of these tanks requires periodic washing. However, waste and heavy oil fractions that build up at the bottom and on the walls of storage tanks are highly viscous and become solid deposits that cannot be removed with conventional pumping. The removal of this material requires washing with solvents and manual cleaning, which is a hazardous, time-consuming, labor intensive, expensive procedure, as cleaning operations may include hot water spraying, solvent liquefaction and subsequent land waste disposal [57,59]. 

The use of microbial biosurfactants is an alternative cleaning procedure to decrease the viscosity of sludge and oil deposits through the formation of an oil-in-water emulsion that facilitates the pumping of waste. Moreover, this process allows the recovery of crude oil when the emulsion is broken. Matsui *et al*. [59] investigated the treatment of oil tank bottom sludge with a novel biosurfactant, JE1058BS, produced by the actinomycete *Gordonia* sp. Dispersion activity was greater than that achieved with a chemical or plant-derived surfactant and the biosurfactant remained stable for at least three weeks. Diab and El Din [60] evaluated the effect of the supernatant from *P.aeruginosa* SH 29 applied to the cleaning of oil-contaminated vessels. The oil was recovered from the bottom and walls of the vessels in just fifteen minutes and floated on the supernatant as a distinct phase. According to the authors, this indicates that the biosurfactant in the sterilized supernatant of *P.aeruginosa* SH 29 can be used directly for cleaning oil storage tanks and other vessels used for the transportation and storage of crude oil. Rocha and Silva *et al*. [6] tested the potential of an isolated biosurfactant from *Pseudomonas cepacia* CCT6659 for cleaning beaker walls contaminated with a layer of oil and found a removal rate of 80%, which suggests the applicability of this biosurfactant in the cleaning of storage tanks.

## 6. Toxicity of (Bio)Surfactants and Dispersants on Organisms in the Bioremediation Process

The toxicity of biosurfactants in the environment is not well known. Edwards *et al*. [61], in a comparison of toxicity of three synthetic surfactants and three microbial surfactants, concluded that the biosurfactants were less toxic than the synthetic surfactants to some invertebrate species. However, the environmental risks posed by biosurfactants, evaluated through microbial community composition analysis have not been sufficiently evaluated [62].

The lower toxicity and higher biodegradability of biological surfactants compared to their chemical counterparts is the main reason for their high acceptability. However, these features are often assumed as only direct consequence of their natural origin. For these reasons, the environmental features of novel biosurfactants should be carefully considered and investigated before their release into the environment [63]. 

While the microbial toxicity of (bio)surfactants is a possible cause of bioremediation inhibition, many (bio)surfactants are not toxic to microorganisms at concentrations near their CMC values [64]. Another possible cause of a reduced rate of bioremediation in the presence of (bio)surfactant is due to increased toxicity of the hydrophobic contaminant due to its increased (pseudo)solubility. (Bio)surfactants increase the apparent aqueous solubility of hydrophobic substrates. In addition, some (bio)surfactants or pseudosolubilized contaminants may exhibit selective toxicity toward specific pure cultures but may have a limited inhibitory impact in a remediation system involving a diverse indigenous microbial population [65].

Regarding the use of dispersants, several classes of these chemical agents are employed for environmental mitigation and cleanup; however, these industrial chemicals may present risks to aquatic organisms individually and when mixed with oil [66,67]. 

The U.S. Clean Water Act and Oil Pollution Act of 1990 requires the maintenance of a National Oil and Hazardous Substances Pollution Contingency Plan (NCP) for response to oil spills that identifies specific commercial products used for control of oil discharges and the quantities and water bodies in which the products may be used [68]. These products consist of dispersants, surface washing agents, surface collecting agents, bioremediation agents, and other miscellaneous oil spill control agents. Under the NCP, the U.S. Environmental Protection Agency has statutory responsibility for obtaining toxicity and efficacy information from the manufacturers before placing a dispersant on the National Product Schedule [67,68]. Fourteen dispersants are listed on the U.S. Environmental Protection Agency (U.S. EPA). Although the exact compositions of most commercially available oil dispersants are proprietary, they typically contain a high percentage of one or more uncharged or charged anionic surfactants of different solubility [68]. 

Probabilistic hazard assessment approaches including Chemical Toxicity Distributions (CTDs) may be useful as an initial step toward prioritizing environmental hazards from the use of dispersants. The CTD approach to two acute toxicity datasets (NCP—the contingency plan dataset and DHOS—a subset of NCP listed dispersants reevaluated subsequent to the Deepwater Horizon oil spill) contain median lethal concentration (LC50) values for dispersants alone and dispersant: oil mixtures, in two standard marine test species, *Menidia beryllina* and *Mysidopsis bahia*. These CTDs suggest that dispersants alone are generally less toxic than oil. In contrast, most dispersant: oil mixtures are more toxic than oil alone [66].

Rigorous toxicological comparison of untreated and dispersant-treated oil is complicated by the fact that when oil, seawater, and dispersants are mixed, a complex multiphase system results. In this complex system, aquatic organisms can be exposed to many toxicants, in many forms, which can have several modes of action. Moreover, chemical dispersion of oil can yield: (1) dissolved petroleum hydrocarbons; (2) dissolved dispersant surfactants; (3) mixed droplets of bulk oil and surfactants (often in micellar form); and (4) nonmicellar, particulate bulk oil [67].

A second important issue for determining the effects of dispersants, is the separate and combined toxicity of the dispersant and the crude oil droplets. Toxicity became an important issue in the late 1960s and early 1970s when application of toxic products resulted in substantial loss of sea life [67]. Since that time, dispersants have been formulated to minimize toxicity to aquatic organisms. For example, the LC50 values of dispersants used in the early 1970s ranged from about 5 to 50 mg/L to the rainbow trout in 96 h exposures. In contrast, LC50s for dispersants available today vary from 200 to 500 mg/L and contain a mixture of surfactants and a less toxic solvent. The U.S. EPA uses a five-step scale of toxicity categories to classify pesticides based on their acute toxicity to aquatic organisms [69]. Nonetheless, use of oil dispersants remains a controversial countermeasure to minimize the impact of oil spills [67,68].

Because the purpose of a dispersant is to facilitate the acceleration of natural attenuation and dilution of spilled oil, the aquatic toxicity of the dispersant: oil mixture is an important consideration. This further complicates a comparative toxicity evaluation, because the course of toxicity in a mixture may be unknown and potentially different for each dispersant. Therefore, although the presence of polycyclic aromatic hydrocarbons does not represent the only factor in determining oil toxicity, evidence links the increased presence of polycyclic aromatic hydrocarbons in chemically dispersed oils to increased toxicity to aquatic organisms [68,70].

The general proposal of toxicity tests is to establish the potential impact of chemicals on the biota of a given environment. The information acquired can be used to regulate use of chemical substances and evaluate the necessity for treatment after their release to the environment [71,72]. An understanding of the factors that contribute to the toxicity of surfactants is necessary to interpret results of toxicity tests of this class of compounds [72]. The most important factor to consider is chemical structure. Since several years, all studies have emphasized the benefits of using rapid, sensitive, reproducible and cost-effective bacterial assays for toxicity screening and assessment [73]. Microorganisms are useful in ecotoxicity testing because they may be evaluated over a short time and they occupy trophic levels in which bioaccumulation and/or bioconcentration are potential problems [74]. Bacterial toxicity tests measure a wide variety of endpoints including mutagenicity tests [75], population growth [76], CO_2_ production [77], enzyme biosynthesis [78], glucose mineralization [79], and bioluminescence inhibition [80].

Several tests have been used to evaluate the toxicity of chemical and biological surfactants on various organisms. The lethal concentration (LC_50_) is a method that evaluates the rate of population mortality of a species and indicates that the higher the concentration, the lower the toxicity of the surfactant [61,81]. The germination index (GI) it is another method which combines measures of relative vegetable seed germination and relative root elongation to evaluate the toxicity of biosurfactants. The GI value of 80% has been used as an indicator of the disappearance of phytotoxicity [6,18,20,22]. 

Table 6 lists toxicity values of (bio)surfactants, dispersants, crude oils and dispersant/crude oil mixtures to vegetables and organisms collected from the literature. 

**Table 6:** Results of toxicity tests of (bio)surfactants, dispersants, crude oils, and dispersant/crude oil mixtures to vegetables and organisms.

Test Compound	Organisms/Vegetables Test	Toxicity	References
**Biosurfactants**			
Emulsan	*Mysidopsis bahia*	LC_50_ (200 mg/L)	[61]
Emulsan	*Menidia beryllina*	LC_50_ (300 mg/L)	[61]
*Candida sphaerica* UCP 0995 biosurfactant	*Brassica oleracea*	86% GI	[81]
*Candida sphaerica* UCP 0995 biosurfactant	*Artemia salina*	LC_50_ (600 mg/L)	[81]
*Candida sphaerica* UCP 0995 biosurfactant	*Brassica oleracea*	no toxicity	[18]
*Candida sphaerica* UCP 0995 biosurfactant	*Artemia salina*	no toxicity	[18]
*Candida lipolytica* UCP 0988 biosurfactant	*Brassica oleracea*	no toxicity	[20]
*Candida lipolytica* UCP 0988 biosurfactant	*Artemia salina*	no toxicity	[20]
*Pseudomonas aeruginosa* UCP 0992 biosurfactant	*Brassica oleracea*	80% GI	[22]
*Pseudomonas aeruginosa* UCP 0992 biosurfactant	*Artemia salina*	LC_50_ (525 mg/L)	[22]
**Emulsifiers/Dispersing agents**			
Dodecylbenzene sulfonate/LAS	*Dugesia japonica*	LC_50_ (1.45 mg/L)	[82]
Lauryl sulfate/SDS	*Dugesia japonica*	LC_50_ (0.36 mg/L)	[82]
Triton X-100	*Mysidopsis bahia*	LC_50_ (3.3 mg/L)	[61]
Triton X-100	*Menidia beryllina*	LC_50_ (2.5 mg/L)	[61]
Lauryl sulfate/SDS	*Americamysis bahia*	LC_50_ (18–23 mg/L)	[68]
Lauryl sulfate/SDS	*Menidia beryllina*	LC_50_ (10 mg/L)	[68]
**Oil spill dispersants**			
Corexit 9500	*Mysidopsis bahia*	LC_50_ (13.4 mg/L)	[61]
Corexit 9500	*Menidia beryllina*	LC_50_ (75.7 mg/L)	[61]
Corexit 9500	*Porites astreoides*	13% surviving	[83]
Corexit 9500	*Montastraea faveolata*	0% surviving	[83]
Corexit 9500	*Americamysis bahia*	42 (mg/L)	[68]
Corexit 9500	*Menidia beryllina*	130 (mg/L)	[68]
Corexit 9500	*Brachionus plicatilis*	LC_50_ (0.447 mg/L)	[67]
Corexit 9500	*Brachionus manjavacas*	LC_50_ (14.2 mg/L)	[67]
**Crude oils**			
BP Horizon source oil	*Porites astreoides*	67% surviving	[83]
BP Horizon source oil	*Montastraea faveolata*	27% surviving	[83]
Louisiana sweet crude oil	*Americamysis bahia*	LC_50_ (2.7 mg/L)	[68]
Louisiana sweet crude oil	*Menidia beryllina*	LC_50_ (3.5 mg/L)	[68]
Macondo sweet crude oil	*Brachionus plicatilis*	LC_50_ (2.47 mg/L)	[67]
Macondo sweet crude oil	*Brachionus* sp.	LC_50_ (19.3 mg/L)	[67]
**Dispersant/oil mixtures**			
Corexit 9500/BP Horizon source oil	*Porites astreoides*	67% surviving	[83]
Corexit 9500/BP Horizon source oil	*Montastraea faveolata*	20% surviving	[83]
Corexit 9500/Louisiana sweet crude oil	*Americamysis bahia*	LC_50_ (5.4 mg/L)	[68]
Corexit 9500/Louisiana sweet crude oil	*Menidia beryllina*	LC_50_ (7.6 mg/L)	[68]
1:10 Corexit 9500/Macondo sweet crude oil	*Brachionus manjavacas*	0.21 (mg/L)	[67]
1:50 Corexit 9500/Macondo sweet crude oil	*Brachionus manjavacas*	0.23 (mg/L)	[67]

GI: germination index; LC_50_: concentration lethal to 50% of the test species.

## 7. Conclusions

This review provided information on the application of biosurfactants as a promising alternative in the petroleum industry and the bioremediation of oil spills. Since biosurfactants are not yet competitive with chemical surfactants from the economic standpoint, a more thorough investigation of biosurfactant production from agro-industrial waste is needed to reduce the production cost and allow the large-scale production of these natural compounds. The versatility and efficiency demonstrated in the application of biosurfactants in the oil production chain and the removal of hydrophobic contaminants make these compounds promising biomolecules.
